# Supplementary material for: The Systems Biology Research Tool: evolvable open-source software
Source: BMC Syst Biol. 2008 Jun 29;2:55. doi: 10.1186/1752-0509-2-55 (PMC2446383; doi:10.1186/1752-0509-2-55)
Supplement: Additional file 1 — SBRT Archive. An archive of the current version of the Systems Biology Research Tool. [file 1752-0509-2-55-S1.zip › sbrt-1.4.0/doc/developers_guide/api/sbrt/shell/text/AbstractLinkedHashSetFormat.html]

AbstractLinkedHashSetFormat


|  |  |  |
| --- | --- | --- |
| **Overview** \| **Package** \| **Class** \| **Use** \| **Tree** \| **Deprecated** \| **Index** \| **Help** | |  |
| PREV CLASS   **NEXT CLASS** | **FRAMES**    **NO FRAMES**     **All Classes** |  |
| SUMMARY: NESTED \| FIELD \| CONSTR \| METHOD | DETAIL: FIELD \| CONSTR \| METHOD |  |


---


## sbrt.shell.text Class AbstractLinkedHashSetFormat<E>

```
java.lang.Object
  sbrt.shell.text.AbstractLinkedHashSetFormat<E>
```

**Type Parameters:**: `E` - the type of element.

**All Implemented Interfaces:**: Formatter<java.util.Set<? extends E>>, LinkedHashSetFormat<E>, Parser<java.util.LinkedHashSet<E>>

**Direct Known Subclasses:**: AmpersandSetFormat, PipeSetFormat, TabSetFormat, WhitespaceSetFormat

---

``` public abstract class AbstractLinkedHashSetFormat<E> extends java.lang.Object implements LinkedHashSetFormat<E> ```

This abstract class is a skeleton implementation of
`LinkedHashSetFormat`. A concrete class with a
defined format can be
created by simply overriding the constructor of this class.

**Author:**
:   This class was written and documented by
    Jeremiah Wright while in the Wagner lab.

---

**Constructor Summary**

`AbstractLinkedHashSetFormat(java.lang.String delimiter,
java.util.regex.Pattern regex,
Formatter<E> elementFormatter,
Parser<E> elementParser)`
  
          Constructs a new `LinkedHashSet` format from
the provided arguments.

`AbstractLinkedHashSetFormat(java.lang.String delimiter,
java.util.regex.Pattern regex,
SimpleFormat<E> elementFormat)`
  
          Constructs a new `LinkedHashSet` format from
the provided arguments.


| **Method Summary** | |
| --- | --- |
| `java.lang.String` | `format(java.util.Set<? extends E> c)`             Returns a formatted string representation of the provided collection. |
| `java.lang.String` | `getDelimiter()`             Returns the delimiter used to separate elements when producing a formatted string. |
| `Formatter<E>` | `getElementFormatter()`             Returns the formatter for the elements of this `LinkedHashSet` format. |
| `Parser<E>` | `getElementParser()`             Returns the parser for the elements of this set format. |
| `java.util.regex.Pattern` | `getRegex()`             Returns the regular expression used to separate elements when parsing a formatted string. |
| `java.util.LinkedHashSet<E>` | `parse(java.lang.String set)`             Parses the provided string and returns its corresponding collection of elements as a `LinkedHashSet`. |

**Methods inherited from class java.lang.Object**

`clone, equals, finalize, getClass, hashCode, notify, notifyAll, toString, wait, wait, wait`

**Methods inherited from interface sbrt.shell.text.Formatter**

`format`

**Constructor Detail**

### AbstractLinkedHashSetFormat

```
public AbstractLinkedHashSetFormat(java.lang.String delimiter,
                                   java.util.regex.Pattern regex,
                                   SimpleFormat<E> elementFormat)
```

:   Constructs a new `LinkedHashSet` format from
    the provided arguments.

    **Parameters:**: `delimiter` - the delimiter used to separate elements of sets when producing a formatted string representation.: `regex` - the regular expression pattern used to parse a string representation of a set with this format. The method call `regex.split(String)` should return an array containing the string representation of the elements of the provided set string.: `elementFormat` - the format used for elements the of sets with this format. **Throws:**: `java.lang.NullPointerException` - if any provided argument is `null`.

---


### AbstractLinkedHashSetFormat

```
public AbstractLinkedHashSetFormat(java.lang.String delimiter,
                                   java.util.regex.Pattern regex,
                                   Formatter<E> elementFormatter,
                                   Parser<E> elementParser)
```

:   Constructs a new `LinkedHashSet` format from
    the provided arguments.

    **Parameters:**: `delimiter` - the delimiter used to separate elements of sets when producing a formatted string representation.: `regex` - the regular expression pattern used to parse a string representation of a set with this format. The method call `regex.split(String)` should return an array containing the string representation of the elements of the provided set string.: `elementFormatter` - the formatter used for elements the of sets with this format.: `elementParser` - the parser used for elements the of sets with this format. **Throws:**: `java.lang.NullPointerException` - if any provided argument is `null`.


**Method Detail**

### getDelimiter

```
public java.lang.String getDelimiter()
```

:   Returns the delimiter used to separate elements
    when producing a formatted string.

    :   **Returns:**: the delimiter used to separate elements when producing a formatted string.

---


### getRegex

```
public java.util.regex.Pattern getRegex()
```

:   Returns the regular expression used to separate
    elements when parsing a formatted string.

    :   **Returns:**: the regular expression used to separate elements when parsing a formatted string.

---


### getElementFormatter

```
public Formatter<E> getElementFormatter()
```

:   Returns the formatter for the elements of this
    `LinkedHashSet` format.

    :   **Specified by:**: `getElementFormatter` in interface `LinkedHashSetFormat<E>`
    :   **Returns:**: the formatter for the elements of this `LinkedHashSet` format.

---


### getElementParser

```
public Parser<E> getElementParser()
```

:   Returns the parser for
    the elements of this set format.

    :   **Specified by:**: `getElementParser` in interface `LinkedHashSetFormat<E>`
    :   **Returns:**: the parser for the elements of this set format.

---


### format

```
public java.lang.String format(java.util.Set<? extends E> c)
```

:   Returns a formatted string representation of the
    provided collection.

    :   **Specified by:**: `format` in interface `LinkedHashSetFormat<E>`
    :   **Parameters:**: `c` - the collection to be formatted. **Returns:**: a formatted string representation of the provided collection.

---


### parse

```
public java.util.LinkedHashSet<E> parse(java.lang.String set)
```

:   Parses the provided string and returns its
    corresponding collection of elements as a
    `LinkedHashSet`.

    :   **Specified by:**: `parse` in interface `LinkedHashSetFormat<E>` **Specified by:**: `parse` in interface `Parser<java.util.LinkedHashSet<E>>`
    :   **Parameters:**: `set` - the string to be parsed. **Returns:**: the provided string and returns its corresponding collection of elements as a `LinkedHashSet`.


---


|  |  |  |
| --- | --- | --- |
| **Overview** \| **Package** \| **Class** \| **Use** \| **Tree** \| **Deprecated** \| **Index** \| **Help** | |  |
| PREV CLASS   **NEXT CLASS** | **FRAMES**    **NO FRAMES**     **All Classes** |  |
| SUMMARY: NESTED \| FIELD \| CONSTR \| METHOD | DETAIL: FIELD \| CONSTR \| METHOD |  |


---
